# Operative and early results of coronary artery bypass grafting in female patients in different body mass indexes

**DOI:** 10.1186/1749-8090-5-119

**Published:** 2010-11-26

**Authors:** Hilmi Tokmakoglu

**Affiliations:** 1 Cardiovascular Surgery Department, Ozel Tekden Hastanesi Kocasinan-Kayseri, Turkey

## Abstract

**Background:**

Female gender has been reported to be an independent risk factor for coronary artery bypass grafting (CABG) in European System for Cardiac Risk Evaluation. The effect of the body size on the CABG outcome is less clear. There is ongoing debate about obesity as a risk factor for adverse outcomes after cardiovascular procedures. The goal of this retrospective study is to evaluate the in hospital and early postoperative outcomes in severe obese, obese and normal-slightly obese female patients after CABG.

**Methods:**

In a four year period a total of 427 female patients underwent isolated CABG under cardiopulmonary bypass. The patients were allocated into three groups according to the Body Mass Index (BMI) as follows; group 1: severe obese patients; BMI > 35, group 2: obese patients; 30≤BMI≤35, group 3: normal-slightly obese patients; BMI < 30

**Results:**

The patients in group 3 were older than the group1 and group 2 (65,6 ± 8,3 year vs 63,01 ± 8,0 and 63,57 ± 8,4 year p < 0,05). In group 1 diabetic patients were more than in group 2 and group 3 respectively (54,4% vs 43,4% and 40%, p < 0,05). Urgent operation was more in group 1 than in group 2 and 3 respectively (37,6% vs 17,2% and 21,2% p < 0,05). The patients in group 3 had significantly greater postoperative drainage at 24 h compared with values in group 1 and group 2 (647 ± 142 ml vs. 539 ± 169 ml and 582 ± 133 ml, p < 0,05). Mortality rate in group 1 was 0,8%, 0% in group 2 and 1,2% in group 3 respectively. Wound problem has occured in 41 patients (9,6%).The percentage of postoperative wound problems was higher in group 1 but did not show statiscially difference. Following discharge a total of 43 (10,1%) patients re-hospitalized within 30 days. Re-hospitalization rate was 16,1% in group1, 9,8% in group 2 and 6,5% in group 3 (p < 0,05).

**Conclusion:**

This study may give an aspect for evaluations of the inhospital-early mortality and morbidity after CABG in female patients in different BMI. Severe obesity is not a risk factor in-hospital mortality in female patients. However, severe obese female patients appear to have more wound problems and re-hospitalization rate after CABG compared to obese and normal-slightly obese patients.

## Background

Female gender has been reported to be an independent risk factor for coronary artery bypass grafting (CABG) in European System for Cardiac Risk Evaluation [1]. In comparison to male patients female patients undergoing CABG have more comorbid risk factors such as; older age, smaller body size, higher prevalance of hypertension, diabetes mellitus, unstable anjina pectoris, smaller size of coronary arteries. Numerous studies have demonstrated increased hospital mortality after CABG in female patients On the other hand studies suggest that female patients clearly benefit from CABG [2-4].

The effect of the body size on the CABG outcome is less clear. There is ongoing debate about obesity as a risk factor for adverse outcomes after cardiovascular procedures [5-7]. Some studies have documented that obesity is not a risk factor for adverse outcomes [8-10] whereas other studies have concluded that extreme obesity is a significant independent predictor for adverse outcomes after CABG [11,12].

The goal of this retrospective study is to evaluate the in hospital and early postoperative outcomes in severe obese, obese and non-obese female patients after CABG.

## Methods

### Patients

In a four year period a total of 427 female patients underwent isolated CABG under cardiopulmonary bypass (CPB). Patients who underwent concomitant procedures such as; valve operation, carotid endarterectomy, were excluded.

### Data collection and definitions

Preoperative, intraoperative and postoperative variables and complications were collected retrospectivly from hospital database. The variables defined as follows; ***diabetes mellitus ***(DM); diet-controlled, oral therapy or insulin dependent DM, ***hypertension***; history of hypertension necessitating medical treatment, ***chronic obstructive pulmonary disease; ***predicting of forced expiratory volume in 1 sec or diffusion capacity less than 75% at pulmonary functional test, ***vascular disease; ***peripheral, abdominal vascular pathology or operation, ***rhythm***; preoperative sinus rhythm, ***ejection fraction ***≤ 40%; determined with preoperative transthoracic echocardiography and named as poor ventricular function, ***pulmonary hypertension; ***pulmonary artey pressure ≥ 30 mmHg determined with preoperative transthoracic echocardiography, ***creatinin level***; blood creatinin level preoperatively and 1st postoperative day, ***thyroid disease; ***hypothyroidism or hyperthyroidism necessitating therapy and all patient's thyroid functions were measured preoperatively, ***left main coronary artery disease ***(LMCA); LMCA stenoses ≥ 70, ***preoperative myocardial infarction ***(MI); history of MI before the operation, ***operative status***; elective: stabil cardiac function and operation is more than 1 day following diagnosis, urgent; the operation that occurred within 24 h of coronary catheterisation because of critically vessel lesion with unstable symptoms, emergency; operation is within the hours for evolving infarction, ischemia not responding to medical therapy, cardiogenic shock or very critically LMCA and right coronary artery disease, ***inotrophic support and/or IABP; ***intraoperative and/or postoperative inotrophic - IABP support due to haemodynamic instability, ***perioperative myocardial infarction; ***a new Q wave and rise in CPK-MB % ≥ 10%, ***re-exploration; ***re-operation for bleeding, tamponade, ***neurological complications; ***postoperative cerebrovascular accidents and/or transient ischemic attack, ***pulmonary complications; ***re-entubation, pulmonary infection, severe athelectazia necessitating intensive fizyotherapy postoperatively, ***wound problems; ***consist in-hospital and within 30 days following discharge; sternotomy and saphenous incision problems were seperately defined as follows; s*ternotomy wound problems*; superficial infections, deep wound-mediastinal involvement and sternal dehiscence, *saphenous incision problems*; wound healing problems requiring surgical debritman, ***mortality; ***all mortality during postoperative hospital stay, ***re-hospitalization; ***following discharge re-hospitalization within 30 days due to pulmonary emboli, deep venous thrombosis, plevral effusion requiring thorasentesis, heart failure, arrhytmia, severe creatinin elevation and wound problems, ***Body Mass Index (BMI); ***calculated as weight (kg)/height squared (m2). The patients were allocated into three groups according to the BMI as follows;

Group 1: Severe obese patients; BMI > 35

Group 2: Obese patients; 30 ≤ BMI ≤ 35

Group 3: Normal-slightly obese patients; BMI < 30

### Surgical technique

All patients were operated using standart CPB tecnique, aortic and two stage right atrial cannulation, systemic hypothermia (28-32 C). Internal thoracic artery and saphenous vein were harvested with conventional technique. Following cross-clamping of the aorta the heart was arrested using intermittant cold blood cardioplegia antegradely and retrogradely, continued with in every 20 min, and finally warm blood cardioplegia was administered before releasing the aortic cross-clamp. The left internal thoracic artery (LITA) was the graft of choice for left anterior descending coronary artery (LAD) in patients and saphenous vein grafts (SVG) for the other anastomosis. After distal anastomoses, proximal anastomoses were done during reperfusion with an partial aortic occluding clamp. During the CPB hematocrit levels were maintained above 20%. Also in all patients efforts were made to ensure perioperative and postoperative blood glucose levels in the range of 150 to 200 mg/dL. After routine closure of the chest, continuous suction (10 mmHg) was applied to the drains, which were milked and stripped at 30-min intervals to ensure tube patency. Chest tubes were removed the following day when the drainage was less than 20 ml/h for consecutive 4 h. All patients were extubated in the intensive care unit (ICU) after establishment of hemodynamic stability. After ICU period, regulation of blood glucose levels were done by internal medicine departmant.

### Statistical analysis

Data evaluation was carried out using a computer statistical package (SPSS 15.0 for Windows, SPSS, Inc., Chicago, IL) and are expressed as means ± SD or as frequencies or percentages. The relationships between independent preoperative and operative variables and postoperative outcome measures were investigated by One-way Anova test or χ^2 ^test for categorical variables. A *P *value of <0.05 was considered significant.

## Results

Preoperative variables are listed in Table 1. There were 125 patients in group 1, 122 patients in group 2 and 170 patients in group 3. The mean BMI was 37,8 ± 2,6 in group1, 32,0 ± 1,3 in group2 and 26,7 ± 2,5 in group3 respectively.

**Table 1:** Preoperative variables

Variable	Group 1 (n:125)	Group 2 (n:122)	Group 3 (n:170)	p-value
Age, (year)	63,01 ± 8,0	63,57 ± 8,4	65,6 ± 8,3	**0,015**
BMI	37,8 ± 2,6	32,0 ± 1,3	26,7 ± 2,5	
DM	54,4%	43,4%	40%	**0,043**
Hypertension	65,6%	62,3%	57,6%	0,3
Smoking	7,2%	11,5%	7,6%	0,4
Cholesterol(mg/dl)	211,2 ± 47,3	209 ± 44,5	208,5 ± 50,2	0,8
COPD	24,8%	13,9%	21,8%	0,08
PVD	4%	3,3%	4,7%	0,8
Sinus rythm	99,2%	99,1%	98,8%	0,5
Thyroid disease				
*Hypothyroidism*	8(6,4%)	6(4,9%)	10(5,9%)	0,8
*Hyperthyroidism*	3(2,4%)	4(3,3%)	6(3,5%)	0,8
PreMI	29,6%	18,2%	22,4%	0,09
Vessel disease				
*1 vessel*	12%	9,8%	11,8%	0,98
*2 vessel*	29,6%	30,3%	29,4%	0,98
*3 vessel*	58,4%	59,8%	58,8%	0,98
*LMCA*	8,8%	8,2%	6,5%	0,7
EF%, mean ± SD	54,3 ± 10,8	55,9 ± 9,6	56,5 ± 10,3	0,17
EF%≤ 40	16,8%	9,8%	10,6%	0,17
PHT (mmHg,%)	16%	14,8%	20,6	0,37
Creatinin1 (mg/dl)	0,89 ± 0,19	0,90 ± 0,15	0,87 ± 0,17	0,26
Creatinin 2 (mg/dl)	1,1 ± 1,2	1,0 ± 0,3	0,95 ± 0,28	0,15

COPD; chronic obstructive pulmonary disease, PVD; peripheral vascular disease, PreMI; preoperative MI, PHT; pılmonary hypertension, Creatinin1; preoperatively, 2; postoperatively creatinin level

The patients in group 3 were older than the group1 and group 2 (65,6 ± 8,3 year vs 63,01 ± 8,0 and 63,57 ± 8,4 year p < 0,05). In group 1 diabetic patients were more than in group 2 and group 3 respectively (54,4% vs 43,4% and 40%, p < 0,05). The remaining factors of hypertension, smoking, cholesterol level, the percantage of chronic obstructive pulmonary disease (COPD), peripheral vascular disease, the percantage of sinus rythm, previous MI, left main coronary artery disease (LMCA), the extension of vessel disease, mean ejection fraction-percantage of EF % ≤ 40, pulmonary hypertension (PHT), mean preoperative and 1 st postoperative day creatinin levels showed no statistical differences between the three groups.

Operative and early postoperative variables are listed in Table 2. Urgent operation was more in group 1 than in group 2 and 3 respectively (37,6% vs 17,2% and 21,2% p < 0,05). Also elective surgery was more in group2 and 3 than in group 1 (75,4% and 75,9% vs 58,4% p < 0,05). The other parameters; mean CABG number, percantage of LITA usage, mean aortic cross clamp time (ACC), cardiopulmonary bypass time (CPBT), percantage of inotrophic support, mean extubation and intensive care unit (ICU) time did not differ beetween the groups. The patients in group 3 had significantly greater postoperative drainage at 24 h compared with values in group 1 and group 2 (647 ± 142 ml vs. 539 ± 169 ml and 582 ± 133 ml, p < 0,05). Four patients in group 3 was revised due to bleeding and/or tamponade whereas none in group 1 and 2. Also occurence of atrial fibrillation (AF), perioperative MI, neurological and pulmonary complications did not differ between the groups. The overall hospital mortality rate was 0,7%. Mortality rate in group1 was 0,8%, 0% in group2 and 1,2% in group3 respectively. Wound problem has occured in 41 patients (9,6%). The percentage of postoperative wound problems was higher in group 1 but did not show statiscially difference. Following discharge a total of 43 (10,1%) patients re-hospitalized within 30 days due to reasons mentioned above. Re-hospitalization rate was 16,1% in group1, 9,8% in group 2 and 6,5% in group 3 (p < 0,05).

**Table 2:** Operative and early postoperative variables

Variable	Group 1	Group 2	Group 3	P-value
Operative status %				
*Emergency*	5,6	6,6	3,5	0,4
*Urgent*	37,6	17,6	21,2	**0,0001**
*Elective*	58,4	75,8	75,9	**0,002**
CABG (n)	2,98 ± 0,89	2,98 ± 0,78	2,95 ± 0,79	0,93
LITA usage%	89,6	90,2	89,7	0,3
ACC (min)	38,3 ± 12,0	37,8 ± 10,1	37,7 ± 10,5	0,71
CPBT (min)	57,6 ± 16,9	56,9 ± 13,6	57,0 ± 14,6	0,82
Inotrophic support %	20	12,3	18,9	0,21
Extubation time(hour)	11,7 ± 3,4	11,7 ± 3,5	12,0 ± 4,4	0,62
ICU time(hour)	25,7 ± 3,6	27,7 ± 24,3	26,7 ± 16,0	0,54
Drenaige (ml)	539 ± 169	582 ± 133	647 ± 142	**0,0001**
Revision	0	0	4(2,6%)	0,053
AF %	24	20,5	20,0	0,68
Perioperative MI	3(2,4%)	3(2,5%)	5(2,9%)	0,9
NC %	0,8	0	0,6	0,63
Pulmonary compl.%	4,8	1,6	2,4	0,41
Wound problems	16(12,8%)	11(9%)	14(8,2%)	0,40
***Sternotomy***				
*Superficial*	4(3,2%)	3(2,5%)	4(2,4%)	0,89
*Deep*	2(1,6%)	1(0,8%)	2(1,2%)	0,85
*Dehisence*	4(3,2%)	3(2,5%)	3(1,8%)	0,72
***Saphenous***	6(4,8%)	4(3,3%)	5(2,9%)	0,67
Mortality	1(0,8%)	0	2(1,2%)	0,49
Re-hospitalization	20(16,1%)	12(9,8%)	11(6,5%)	**0,02**
*DVT*	3	0	0	
*DVT+PE*	1	1	0	
*Plevral effusion*	2	1	3	
*Heart failure*	2	1	*0*	
*Arrythmia*	2	2	1	
*Creatinin elevation*	2	2	1	
*Wound problems*	*8*	5	6	

ACC; aortic cross clamp time, CPBT; cardiopulmonary bypass time, ICU; intensive care unit, AF; atrial fibrillation, NC; neurological complication, DVT; deep venos thyrombosis, PE; pulmonary emboli

## Discussion

Cardiovascular disease is leading cause of morbidity and mortality for women in developed and developing countries. There is considerable evidence that female gender carry a higher CABG mortality when compared with the male patients [13-15]. On the other hand obesity is considered to be a major risk factor in patients undergoing CABG. With the increasing of BMI also comorbidty increases [16,17]. There are major differences in the risk profile of female patients compared with the profile of male patients [18,19]. The great majority of studies show that diabetes is 40% to 50% more common in female patients than male patients undergoing CABG [20,21]. In this study diabetes is found to be 45,3% in totally whereas 54,4% in severe obese patients. It is well-known that there is a clear association of diabetes with adverse postoperative outcome in surgical patients. Despite the usage of prophylactic antibiotics, sternal wound infections are associated high mortality and morbidity. In our severe obese patients sternal superficial infections and sternal dehisence were more common but not statistically significant than the other groups. Some studies were emphasised that hyperglycemia in the first 2 postoperative days is the single most important predictor of mediastinitis after cardiac surgery and blood glucose level must be maintained below 200 mg/dL [22,23]. As mentioned before in our patients special efforts were made to ensure perioperative and postoperative blood glucose levels in the range of 150 to 200 mg/dL with the using of continuous intravenous insulin infusions.

Hypothyroidism is associated with impaired ventricular contractility and in female patients there is a higher incidence of hypothyroidism undergoing CABG [24]. In the study of Zindrou and colleagues they found a high mortality rate (16,7%) in female patients requiring thyroid replacement therapy whereas not in male patients [25]. In our clinic all patient's thyroid functions were measured preoperatively and hypothyroidic patients were maintained in a euthyroid state before the operation. In non-elective status patients therapy was begun before the operation and contiuned following operation.

The use of at least one LITA confers both in-hospital and long-term improvement in CABG mortality [26,27]. However usage of LITA as a conduit in female patients is only 60%-75% of cases [28,29]. This is significantly less than LITA usage in male patients. Actually there is no objective reason to use the LITA less ferquently in females than the males. Perhaps the presence of a soft friable sternum that predisposes sternal dehisence is a valid reason to avoid use of LITA [30]. In the study of Aldea and colleagues LITA was used in 91% of female patients and found no gender differences in operative mortality [31].

In most series there is a higher rate of non-elective CABG in female patients [28,31]. Likewise in our study the rate of non-elective surgery was 29,2% in all patients whereas statiscially higher in group 1 than the other groups. Also in other studies it was emphasised that use of LITA is safe when urgent and emergency operations are being performed [32,33]. In our study use of LITA as a conduit was found 89,6% in severe obese group even percentage of urgent surgery was high.

Some studies found a significant reduction for the risk of postoperative bleeding in obese patients [34,35]. Likewise in our study the amount of bleeding and re-exploration rate was less in obese grups than the non-obese grup.

Atrial fibrillation (AF) is a frequent event after CABG with an incidence of 15-40%. It may result in hemodynamic compromise during the postoperative period. There are some reports saying AF are seen in high BMI score patients [36,37]. In our patients there was no significant difference between the three groups

We did not find a significant difference ICU time, creatinin levels, neurological complications and mortality rates between the three groups.

Obesity alters the pulmonary function leading to an increase in functional residual capacity, and a decrease in vital capacity and maximum voluntary ventilation [38]. In addition, anaesthetic drugs that are revealed from the fat tissue may prolong the entubation time. Also patients with low BMI have remarkable haemodilution, fall in the oncotic pressure during CPB and this may lead excess fluid extravasation [39]. In this study we did not find a significant difference for extubation time between the groups. Hovewer postoperatively pulmonary complication was more common in group1 but showed no statiscially difference.

Readmission following discharge is an important adverse outcome of CABG surgery. Hannan El et al. examined the frequency and causes of CABG surgery readmissions and in their study they found 15,3% readmisions within 30 days following discharge. Also they found female gender is a risk factor of readmission after CABG [40]. In our study a total of 43 (10,1%, mostly in group1) patients readmitted and re-hospitalized following discharge.

## Limitations of the study

This study was done on a retrospective series from a single institution and also gives only in-hospital and early postoperative period outcomes. Further complementary studies with higher number of patients and including early, mid-term, long-term results in contemporary methods are warranted.

## Conclusion

This study may give an aspect for evaluations of the inhospital-early mortality and morbidity after CABG in female patients in different BMI. Female gender and also severe obesity is not a risk factor in-hospital mortality. However, severe obese female patients appear to have more wound problems and re-hospitalization rate after CABG compared to obese and non-obese patients.

## Competing interests

The authors declare that they have no competing interests.

## Authors' contributions

HT: Performed operations, wrote manuscript.

Author read and approved the final manuscript.

## References

[B1] Roques F Nashef SA Michel P Gauducheau E de Vincentiis C Baudet E Risk factors and outcome in European cardiac surgery: analysis of the EuroSCORE multinational database of 19030 patients Eur J Cardiothoracic Surg 1999 15 816 822 10.1016/S1010-7940(99)00106-2 10431864

[B2] Loop FD Golding LR MacMillan JP Cosgrove DM Lytle BW Sheldon WC Coronary artery surgery in women compared with men: analysis of risk and long term results J Am Coll Cardiol 1983 1 383 390 10.1016/S0735-1097(83)80064-3 6600758

[B3] Gardner TJ Horneffer PJ Gott VL Watkins L Jr Baumgartner WA Borkon AM Coronary artery bypass grafting in women Ann Surg 1985 201 780 784 10.1097/00000658-198506000-00016 3873919 PMC1250819

[B4] Herlitz J Brandrup-Wognsen G Karlson BW Sjoland H Karlsson T Caidahl K Mortality, risk indicators of death, mode of death and symptoms of angina pectoris during5 years aftercoronary artery bypass grafting in men and women J Intern Med 2000 247 500 506 10.1046/j.1365-2796.2000.00645.x 10792565

[B5] Jin R Grunkemeier GL Furnary AP Handy JR Jr Is obesity a risk factor for mortality in coronary artery bypass surgery? Circulation 2005 111 3359 3365 10.1161/CIRCULATIONAHA.104.489880 15967852

[B6] Pan W Hindler K Lee V Vaughn W Collard CD Obesity in diabetic patients undergoing coronary artery bypass graft surgery is associated with increased postoperative morbidity Anesthesiology 2006 104 441 7 10.1097/00000542-200603000-00010 16508390

[B7] Prabhakar G Haan CK Peterson ED Coombs LP Cruzzavala JL Murray GF The risks of moderate and extreme obesity for coronary artery bypass grafting outcomes: A study from The Society of Thoracic Surgeons' Database Ann Thorac Surg 2002 74 1125 1131 10.1016/S0003-4975(02)03899-7 12400756

[B8] Moulton MJ Creswell LL Mackey ME Cox JL Rosenbloom M Obesity is not a risk factor for significant adverse outcomes after cardiac surgery Circulation 1996 95 Suppl 9 1187 1192 8901725

[B9] Fisher LD Kennedy JW Davis KB Maynard C Fritz JK Kaiser G Myers WO Association of sex, physical size, and operative mortality after coronary artery bypass in the coronary artery study (CASS) J Thorac Cardiovasc Surg 1982 84 334 341 6981033

[B10] Prasad US Walker WS Sang CTM Campenella C Cameron EWJ Influence of obesity on the early and long term results of surgery for coronary artery disease Eur J Cardiothorac Surg 1991 5 67 73 10.1016/1010-7940(91)90003-3 2018657

[B11] Prabhakar G Haan CK Peterson ED Coombs LP Cruzzavala JL Murray GF The risks of moderate and extreme obesity for coronary artery bypass grafting outcomes: A study from The Society of Thoracic Surgeons' Database Ann Thorac Surg 2002 74 1125 1131 10.1016/S0003-4975(02)03899-7 12400756

[B12] Habib RH Zacharias A Schwann TA Effects of obesity and small body size on operative and long-term outcomes of coronary artery bypass surgery: a propensity-matched analysis Ann Thorac Surg 2005 79 1976 1986 10.1016/j.athoracsur.2004.11.029 15919295

[B13] Carey JS Cukingnan RA Singer LKM Health status after myocardial revascularization: inferior status in women Ann Thorac Surg 1995 59 112 1117 10.1016/0003-4975(94)00702-9 7818310

[B14] Edwards FH Carey JS Grover FL Bero JW Hartz RS Impact of gender on coronary bypass operative mortality Ann Thorac Surg 1998 66 125 131 10.1016/S0003-4975(98)00358-0 9692451

[B15] Hammar N Sandberg E Larsen FF Ivert T Comparison of early and late mortality in men and women after isolated coronary artery bypass graft surgery in Stockholm, Sweden1980 to 1989 J Am Coll Cardiol 1997 29 659 664 10.1016/S0735-1097(96)00531-1 9060908

[B16] Health counsil of the Netherlands Overweight and obesity 2003 The Hague: Health counsil of the Netherlands Publication no. 2003/07

[B17] Ray CS Sue DY Bray G Hansen JE Wasserman K Effects of obesity on respiratory function Am Rev Respir Dis 1983 128 501 506 6614644 10.1164/arrd.1983.128.3.501

[B18] Zitser-Gurevich Y Simchen E Galai N Mandel M Effect of perioperative complications on excess mortality among women after coronary bypass J Thorac Cardiovasc Surg 2002 123 The Israeli Coronary Bypass Graft study(ISCAB) 517 524 10.1067/mtc.2002.120012 11882825

[B19] Koch CG Khandwala F Nussmeier N Blackstone EH Gender profiling in coronary artery bypass grafting J Thorac Cardiovasc Surg 2003 126 2044 2051 10.1016/S0022-5223(03)00955-3 14688724

[B20] Vaccarino V Abramson JL Veledar E Weintraub WS Sex differences in hospital mortality after coronary artery bypass surgery Circulation 2002 105 1176 1181 10.1161/hc1002.105133 11889010

[B21] Woods SE Noble G Smith JM Hasselfeld K The influence of gender in patients undergoing coronary artery bypass graft surgery: an eight year prospective hospitalized cohort study J Am Coll Surg 2003 196 428 434 10.1016/S1072-7515(02)01756-8 12648695

[B22] Furnary AP Zerr KJ Grunkemeier G Starr AS Continuous intravenous insulin infusion reduces the incidence of deep sternal wound infection in diabetic patients after cardiac surgical procedures Ann Thorac Surg 1999 67 352 360 10.1016/S0003-4975(99)00014-4 10197653

[B23] Zerr KJ Furnary AP Grunkemeier GL Glucose control lowers the risk of wound infection in diabetics after open heart operations Ann Thorac Surg 1997 63 356 361 10.1016/S0003-4975(96)01044-2 9033300

[B24] Eagle KA Guyton RA Davidoff R ACC/AHA 2004 guideline update for coronary artery bypass graft surgery: a report of the American College of Cardiology/American Heart Association Task Force on Practice Guidelines (Committee to Update the 1999 Guidelines for Coronary Artery Bypass Graft Surgery) J Am Coll Cardiol 2004 44 1146 54 10.1016/j.jacc.2004.07.021 15337239

[B25] Zindrou D Taylor KM Bagger JP Excess coronary artery bypass mortality among women with hypothyroidism Ann Thorac Surg 2002 74 2121 2125 10.1016/S0003-4975(02)04082-1 12643405

[B26] Edwards FH Clark RE Schwartz M The impact of internal mammary artery conduits on operative mortality in coronary revascularization Ann Thorac Surg 1994 57 27 32 10.1016/0003-4975(94)90360-3 7904145

[B27] Leavitt BJ O'Connor GT Olmstead EM Use of the internal mammary artery graft and in-hospital mortality associated with coronary artery bypass grafting Circulation 1998 98 130 10.1161/01.cir.103.4.507 11157714

[B28] Abramov D Tamariz MG Sever JY The influence of gender on the outcome of coronary artery bypass surgery Ann Thorac Surg 2000 70 800 806 10.1016/S0003-4975(00)01563-0 11016313

[B29] Lawton JS Brister SJ Petro KR Dullum M Surgical revascularization in women: unique intraoperative factors and considerations J Thorac Cardiovasc Surg 2003 126 936 938 10.1016/S0022-5223(03)00805-5 14566226

[B30] Mickleborough LL Takagi Y Mariyama H Sun Z Mohamed S Is sex a factor in determining operative risk for aortocoronary bypass surgery? Circulation 1995 92 supp 1 1180 1184 10.1161/01.cir.92.9.80 7586467

[B31] Aldea GS Gaudiani JM Shapira OM Effect of gender on postoperative outcomes and hospital stays after coronary artery bypass grafting Ann Thorac Surg 1999 67 1097 1103 10.1016/S0003-4975(99)00055-7 10320257

[B32] Cohn LH Use of the internal mammary artery graft and in-hospital mortality and other adverse outcomes associated with coronary artery bypass surgery Circulation 2001 103 483 484 11157709 10.1161/01.cir.103.4.483

[B33] Leavitt BJ O'Connor GT Olmstead EM Use of the internal mammary artery graft and in-hospital mortality and other adverse outcomes associated with coronary artery bypass surgery Circulation 2001 103 507 512 11157714 10.1161/01.cir.103.4.507

[B34] Birkmeyer NJO Charlesworth DC Hernandez F Leavitt BJ Marrin CA Morton JR Olmstead EM O'Connor GT Obesity and risk of adverse outcomes associated with coronary artery bypass surgery Circulation 1998 97 1689 1694 9591762 10.1161/01.cir.97.17.1689

[B35] Engelman DT Adams DH Byrne JG Aranki SF Collins JJ Couper GS Allred EN Cohn LH Rizzo RJ Impact of body mass index and albumin on morbidity and mortality after cardia surgery J Thorac Cardiovasc Surg 1999 118 867 873 10.1016/S0022-5223(99)70056-5 10534692

[B36] Moulton MJ Creswell LL Mackey ME Cox JL Rosenbloom M Obesity is not a risk factor for significant adverse outcomes after cardiac surgery Circulation 1996 95 Suppl 9 1187 1192 8901725

[B37] Koshal A Hendry P Raman SV Keon WJ Should obese patients not undergo coronary artery surgery? Can J Surg 1985 28 331 334 3874678

[B38] Jenkins SC Moxham J The effects of mild obesity on lung function Respir Med 1991 85 309 311 10.1016/S0954-6111(06)80102-2 1947368

[B39] Reeves BC Ascione R Chamberlain MH Angelini GD Effect of body mass index on early outcomes in patients undergoing coronary artery bypass surgery J Am Coll Cardiol 2003 42 668 676 10.1016/S0735-1097(03)00777-0 12932599

[B40] Hannan EL Racz MJ Walford G Predictors of readmission for complications of coronary artery bypass surgery JAMA 2003 290 773 780 10.1001/jama.290.6.773 12915430

